# Engagement of α_3_β_1_ and α_2_β_1_ integrins by hypervirulent *Streptococcus agalactiae* in invasion of polarized enterocytes

**DOI:** 10.3389/fmicb.2024.1367898

**Published:** 2024-03-06

**Authors:** Giuseppe Valerio De Gaetano, Germana Lentini, Francesco Coppolino, Agata Famà, Giampiero Pietrocola, Concetta Beninati

**Affiliations:** ^1^Department of Human Pathology, University of Messina, Messina, Italy; ^2^Department of Biomedical, Dental and Imaging Sciences, University of Messina, Messina, Italy; ^3^Department of Molecular Medicine, Biochemistry Section, University of Pavia, Pavia, Italy; ^4^Scylla Biotech Srl, Messina, Italy

**Keywords:** group B streptococcus, intestinal epithelium, Caco-2 cells, bacterial invasion, bacterial adherence

## Abstract

The gut represents an important site of colonization of the commensal bacterium *Streptococcus agalactiae* (group B Streptococcus or GBS), which can also behave as a deadly pathogen in neonates and adults. Invasion of the intestinal epithelial barrier is likely a crucial step in the pathogenesis of neonatal infections caused by GBS belonging to clonal complex 17 (CC17). We have previously shown that the prototypical CC17 BM110 strain invades polarized enterocyte-like cells through their lateral surfaces using an endocytic pathway. By analyzing the cellular distribution of putative GBS receptors in human enterocyte-like Caco-2 cells, we find here that the alpha 3 (α_3_) and alpha 2 (α_2_) integrin subunits are selectively expressed on lateral enterocyte surfaces at equatorial and parabasal levels along the vertical axis of polarized cells, in an area corresponding to GBS entry sites. The α_3_β_1_ and α_2_β_1_ integrins were not readily accessible in fully differentiated Caco-2 monolayers but could be exposed to specific antibodies after weakening of intercellular junctions in calcium-free media. Under these conditions, anti-α_3_β_1_ and anti-α_2_β_1_ antibodies significantly reduced GBS adhesion to and invasion of enterocytes. After endocytosis, α_3_β_1_ and α_2_β_1_ integrins localized to areas of actin remodeling around GBS containing vacuoles. Taken together, these data indicate that GBS can invade enterocytes by binding to α_3_β_1_ and α_2_β_1_ integrins on the lateral membrane of polarized enterocytes, resulting in cytoskeletal remodeling and bacterial internalization. Blocking integrins might represent a viable strategy to prevent GBS invasion of gut epithelial tissues.

## Introduction

*Streptococcus agalactiae* or group B streptococcus (GBS) is a frequent colonizer of the human intestine that can also cause a wide range of diseases, including sepsis, meningitis, endocarditis, soft tissue infections and arthritis. Infections affecting adults, including patients with chronic underlying disease (such as diabetes and solid tumors) and the elderly, are steadily increasing (Farley, 2001; Sambola et al., 2002; Zi et al., 2022; Keogh and Doran, 2023). Moreover, in the neonate, GBS is a highly prevalent cause of serious disease including sepsis, meningoencephalitis, and permanent neurological disorders (Edwards and Baker, 2005; Verani et al., 2010). Based on pathogenesis, clinical features and properties of infecting strains, neonatal infections can be grouped into two different disease types. Early-Onset Disease (EOD) manifests itself during the first week of life, while Late-Onset Disease (LOD) occurs from day 8 to 89 after birth (Melin, 2011; Karampatsas et al., 2022; Lohrmann et al., 2023). For EOD, the prevalent route of GBS transmission to the newborn is inhalation of contaminated amniotic fluid or secretions from the genital tract during parturition (Edwards and Baker, 2005; Melin, 2011). In the case of LOD, GBS can be acquired at the time of delivery or postnatally via maternal sources, such as breast milk, or by horizontal transmission. Colonization of the intestine and invasion of the intestinal barrier are considered important events in many cases of LOD. For example, intestinal colonization can be detected for up to 3 months in 40% of infants that become colonized at birth (Weindling et al., 1981). Many LODs, particularly meningitis cases, are caused by strains of capsular serotype III belonging to clonal complex 17 (CC17), which, for this reason, has been dubbed hypervirulent GBS (Lamy et al., 2006; Poyart et al., 2008; Tazi et al., 2010; Bellais et al., 2012). Identification of the bacterial and host factors governing interactions of these bacteria with intestinal cells seems important to understand the pathogenesis of LOD and develop alternative strategies to prevent this condition. However, little is known of the mechanisms by which hypervirulent GBS colonize the human intestine.

Pathogens interact with intestinal epithelial cells by means of different virulence factors, mainly adhesins, to colonize mucosal tissues and often disseminate to distant sites. GBS expresses a complex set of components capable of interacting with different cell receptors and components of the extracellular matrix (ECM) (Pietrocola et al., 2018). In the intestinal mucosa, the apical and basolateral surfaces of polarized enterocytes express different sets of receptors, due to the distinctive distribution of membrane proteins involved in maintenance of cell polarity and intercellular junctions (Groschwitz and Hogan, 2009; Chelakkot et al., 2018). One of the main functions of intercellular tight junctions is to reduce potential interactions between basolateral cell surfaces and luminal pathogens (Ulluwishewa et al., 2011; Gieryńska et al., 2022), even though these junctions represent themselves a frequent microbial target (Paradis et al., 2021). Moreover, microbial toxins or inflammatory responses can weaken intercellular junctions, leading to bacterial entry through the lateral surfaces of intestinal epithelial cells, as shown for *Listeria monocytogenes, Shighella flexneri*, and *Campylobacter jejuni* (Mounier et al., 1992; Gaillard and Finlay, 1996; Bouwman et al., 2013). Previous studies have shown that GBS can transiently open the tight junctions of Caco-2 enterocyte-like cell and cervical epithelial cells and reach the paracellular space (Soriani et al., 2006). Once in this location, GBS selectively adhere to lateral cell membranes at parabasal sites, from which they invade cells through a caveolae-dependent endocytotic pathway (De Gaetano et al., 2021). Collectively, these data suggest that, similar to other luminal pathogens, GBS can recognize receptors distributed in the lateral surfaces of polarized intestinal cells that are hidden when the monolayer is intact, but that become accessible under conditions that weaken intercellular junctions, such as toxin release, inflammatory reactions or reduced extracellular Ca^++^ concentrations (De Gaetano et al., 2021).

Integrins are cell surface glycoproteins belonging to a large family of αβ heterodimeric transmembrane receptors which mediate cell-cell and cell-ECM interactions in various tissues, including the intestine (Hynes, 2002). In humans, there are 18 different α subunits and 8 β subunits which make up 24 different receptors each containing an extracellular, a transmembrane and a cytoplasmic region (Frantz et al., 2010). Integrin-surface expression and functions vary in different cell lineages. In tissue cells, binding of integrins to ECM components results in the rapid recruitment of cytoskeletal proteins and in the formation of anchoring points to the surrounding ECM (van der Flier and Sonnenberg, 2001; Brunton et al., 2004).

Several pathogens use integrins and integrin-dependent intracellular signaling for their adhesion to and invasion of host cells (Scibelli et al., 2007; De Gaetano et al., 2022). In this process, pathogens can directly interact with integrins or use other host molecules, such as ECM components, as a bridge. Previous studies have demonstrated both direct and indirect interactions of GBS with integrins. For example, these bacteria can invade respiratory cells by using the Plasminogen binding surface Protein (PbsP) cell wall adhesin to bind vitronectin, which acts as a bridge to activate the α_v_ integrins (Buscetta et al., 2016; De Gaetano et al., 2018; Lentini et al., 2018; Coppolino et al., 2021). Direct GBS-integrin interactions have also been documented (Cuzzola et al., 2000; Bolduc and Madoff, 2007). Hypervirulent GBS were recently found to bind integrins α_5_β_1_ and α_v_β_3_ via the Srr2 adhesin and invade by this mechanism brain endothelial cells (Deshayes de Cambronne et al., 2021). The present study was undertaken to ascertain whether integrins are involved in GBS invasion of enterocytes. To this end, we used the Caco-2 cells to model the intestinal epithelial barrier because of their resemblance to mature enterocytes after a process of spontaneous cell polarization involving the development of intercellular junctions and an apical brush border (Ramond et al., 1985; Delie and Rubas, 1997; De Gaetano et al., 2021).

## Materials and methods

### Bacterial strain and reagents

The GBS strain BM110 (serotype III, CC17; Pellegrini et al., 2022) was used in the present study. This strain was grown at 37°C in Todd-Hewitt broth (THB, Difco Laboratories) until the mid log phase. *Staphylococcus aureus* strain Newman was grown in Tryptic Soy Broth (TSB; Oxoid) at 37°C and used as a control in some experiments. Rabbit polyclonal anti-GBS serum has been previously described (De Gaetano et al., 2021). Rabbit anti-α_3_ polyclonal (21992-1-AP, Proteintech), mouse anti-α_2_ monoclonal (14-0498-80, Invitrogen), rabbit anti-β_1_ polyclonal (12594-1-AP, Proteintech) and mouse anti-integrin α_5_β_1_ monoclonal (MAB1969, Millipore) antibodies were obtained commercially, while rabbit anti-α_5_, -α_1_, -α_V_, and -β_3_ polyclonal antibodies were a generous gift from Dr. G. Tarone (University of Turin, Italy) and have been previously described (Pietrocola et al., 2015; De Gaetano et al., 2022). For immunofluorescence analysis, Alexa Fluor 488-conjugated anti-rabbit IgG (ab15007) and anti-mouse FITC secondary (ab6785) antibodies, both from Abcam, were used. For enzyme-linked assays (ELISAs), anti-rabbit (A3687, Sigma) and anti-mouse (Biorad 170-6520) alkaline-phosphatase (AP) conjugated secondary antibodies were used.

### Caco-2 cultures

The human enterocyte-like Caco-2 cell line (ATCC HTB-37; colorectal adenocarcinoma) was seeded in 24-well plates at a density of 1 × 10^4^ cells per well in Roswell Park Memorial Institute (RPMI) 1640 medium (R8758, Sigma-Aldrich) supplemented with 10% (vol/vol) fetal bovine serum (FBS, 1203C, Sigma-Aldrich) and penicillin-streptomycin (100 IU of penicillin and 0.1 mg/ml of streptomycin, P433, Sigma-Aldrich) and cultured at 37°C in a humidified 5% CO_2_ incubator, as previously described (De Gaetano et al., 2021). The culture medium was changed every 3 days. Confluence and cellular differentiation were monitored by optical microscopy and immunofluorescence. Five-day-old and 21-day-old Caco-2 cells were used in the present study.

### Enzyme-linked assay

Integrin subunit expression was determined in ELISA assays in 21-day-old Caco-2 monolayers after treatment with ethylene glycol-bis-(β-aminoethyl ether)-*N, N*, *N*′, *N*′-tetraacetic acid (EGTA) (E3889, Sigma-Aldrich) at a 10 μM concentration in Dulbecco's phosphate buffered saline without calcium and magnesium (DPBS; Euroclone S.p.A., Milan, Italy) for 15 min. Briefly, EGTA-treated cell monolayers were washed with DPBS and fixed with 3.7% formaldehyde for 15 min at room temperature (RT). Subsequently, enterocytes were washed and blocked with 2% bovine serum albumin (BSA) in DPBS for 2 h at RT with gentle agitation. All antibodies against integrin subunit were diluted 1:1,000 in 1% BSA and incubated with enterocytes for 1 h. Removal of excess of anti-integrin antibodies with several washes was followed by an additional 1 h of incubation with AP-conjugated secondary antibodies diluted 1:10,000 in 1% BSA. After the addition of para-nitrophenyl phosphate (pNPP), absorbance at 405 nm was determined using a plate reader. Data concerning integrin expression in EGTA-treated cell monolayers were expressed as fold changes compared to untreated monolayers.

### Adhesion and invasion assays

Adhesion and invasion assays were performed as previously described (De Gaetano et al., 2021; Lentini et al., 2022). Briefly, bacteria were grown to the mid-log phase, collected by centrifugation, washed and suspended to the concentration of ~1–2 × 10^6^ CFU/ml in pre-warmed serum-free RPMI. Caco-2 cells were washed three times and bacteria were added to 5-day-old sub-confluent islands or 21-day-old post-confluent monolayers at a MOI of ~35. After a 1 h incubation at 37°C in the presence of 5% CO_2_, extracellular bacteria were washed away by rinsing cultured cells three times with DPBS. The numbers of adhering bacteria to the cells were measured by plating cell lysates on blood agar plates. To measure bacterial internalization, the infected cells were washed three times with DPBS and incubated for one additional hour in RPMI containing bactericidal amounts of penicillin and streptomycin (200 U/ml and 200 μg/ml, respectively), as previously described (Famà et al., 2020; Lentini et al., 2020). We found in preliminary experiments that these antibiotic treatment conditions result in elimination of all extracellular bacteria, as visualized using anti-GBS antibodies in non-permeabilized cells. To investigate the role of integrins, enterocytes were treated for 30 min with different concentrations (0.6, 3, and 15 μM in RPMI) of tri-peptides with an arginine-glycine-aspartic (RGD, Sigma-Aldrich, St. Louis, Missouri, U.S.A.) or arginine-glycine-glutamic acid (RGE, Sigma-Aldrich, St. Louis, Missouri, U.S.A.) sequence, the latter being used as a control. To study the involvement of integrin subunits in GBS interactions with enterocytes, cells were pre-incubated with antibodies against α-, β- or both integrin subunits for 30 min before infection. Antibodies were diluted at ratios ranging from 1:1,000 to 1:5,000 in RPMI, as indicated in the Figures and Figure legends. The effects of anti-integrin antibodies were evaluated in comparison with normal IgG used as a control. In studies employing mature 21-day-old monolayers, temporary weakening of intercellular junctions was achieved with EGTA as described above under “enzyme-linked assay”. Adherence and invasion were expressed as percentage values using the formula: recovered CFU/initial inoculum CFU × 100.

### Fluorescence microscopy

Structured-illumination fluorescence microscopy analysis was performed using a Zeiss Observer.Z1 Apotome apparatus and images were acquired with the AxionVision software, as previously described (De Gaetano et al., 2021). Briefly, Caco-2 cells were cultured in coverslips and their confluence was monitored daily. To visualize bacterial invasion, cells were infected for 1 h, washed and incubated for a further 1 h time in the presence of penicillin (200 U/ml) and streptomycin (200 μg/ml) to remove residual extracellular bacteria. Then coverslips were washed several times, fixed with 3.7% formaldehyde for 15 min at RT and permeabilized with 0.1% Triton X-100 for 10 min. After a 1 h blocking step with BSA (2% in DPBS), infected cells were incubated for 1 h with rabbit polyclonal anti-GBS serum followed by Alexa Fluor 488-conjugated secondary anti-rabbit antibody, both diluted 1:2,000 in DPBS supplemented with BSA 1%. To visualize the surface expression of integrin subunits, enterocytes were fixed and stained with anti-integrin antibodies diluted 1:2,000 with 1% BSA in DPBS. Labeled secondary antibodies (1:2,000 in 1% BSA in DPBS) were used according to the anti-integrin antibody producing species. The contribution of integrin subunits to GBS internalization was studied after permeabilization of fixed coversplis, as described above. Phalloidin-iFluor 555 (ab176756, Abcam) and 40-6-diamidino-2-phenylindole (DAPI) (D8417, Sigma-Aldrich) were used according to the manufacturer's instructions to label, respectively, actin and DNA (both eukaryotic and bacterial).

### Statistical analysis

All experiments were repeated at least three times and the data were expressed as means ± standard deviations (SD). Data were analyzed for statistical significance by the ANOVA test followed by the Bonferroni correction for multiple comparisons or by the Mann-Whitney test. A *p*-value of 0.05 was used as the threshold for significance.

## Results

### Expression of integrins in human enterocyte-like cells

**Figure 1:**
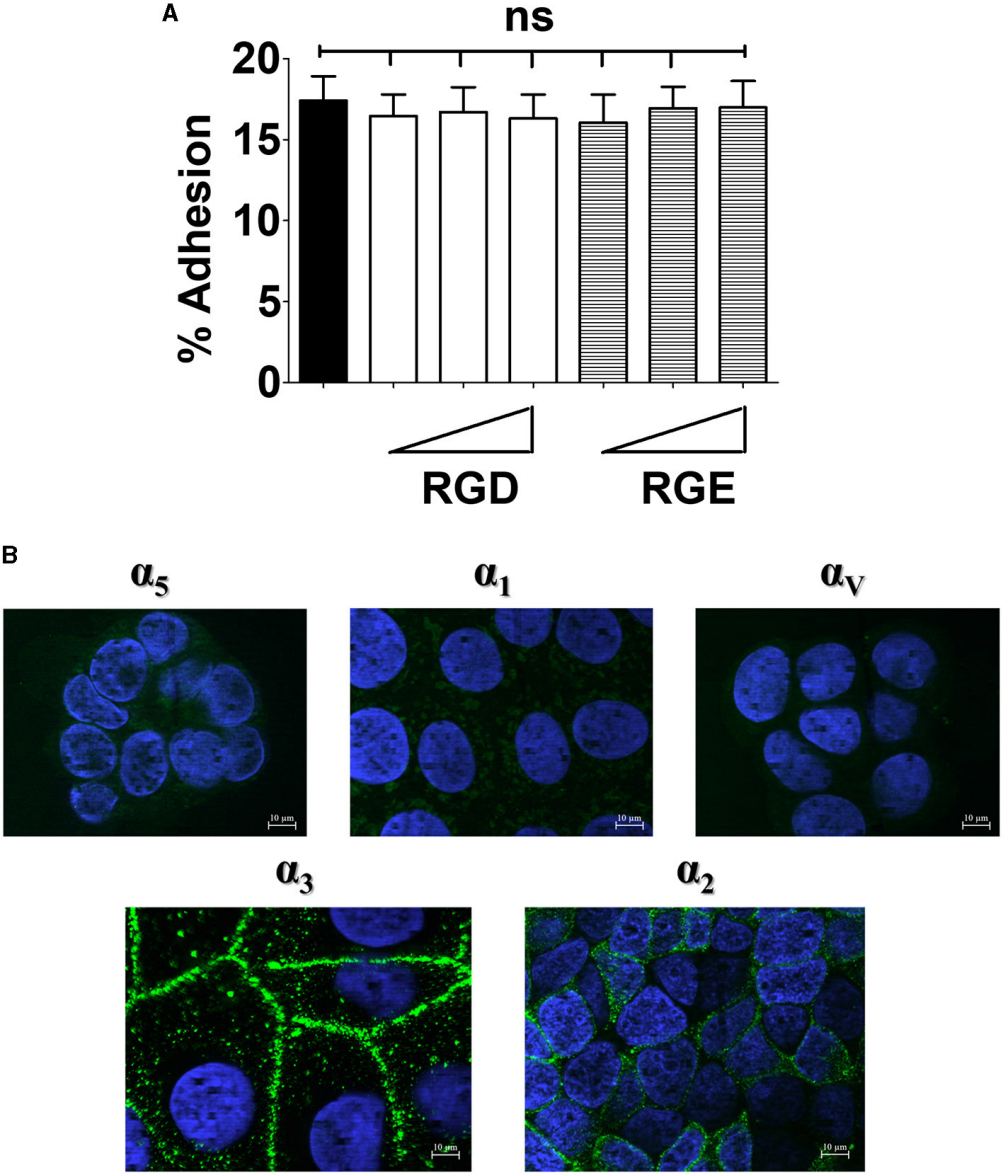
Effects of the RGD peptide on GBS adhesion and expression of integrins in enterocyte-like cells. **(A)** Five-day-old Caco-2 cells were pre-treated with increasing concentrations (ranging from 0.6 to 15 μM) of RGD or RGE tri-peptides and infected with GBS. Streptococcal adherence is expressed as the percentage of input bacteria measured in cell lysates. Shown are means ± SD of five independent experiments conducted in triplicate. ns, non-significant by the ANOVA test followed by the Bonferroni correction. **(B)** Fixed, unpermeabilized 5-day-old Caco-2 cells were probed with antibodies specific for α_5_-, α_1_-, α_V_-, α_3_-, and α_2_-integrin subunits, followed by AlexaFluor 488- or FITC-labeled secondary antibodies (green), and analyzed by fluorescence microscopy. Cell nuclei were stained in blue with DAPI. Scale bar = 10 μm. Shown are representative images from three independent experiments.

The RGD amino acid motif (Arg-Gly-Asp) is present in several extracellular matrix glycoproteins, such as fibronectin and vitronectin, and can act as an anchoring point for integrins expressed on the cell surface (Takahashi et al., 2007; Singh et al., 2010). Based on these interactions, the integrin family can be classified into RGD-recognizing (e.g., α_V_β_3_, α_V_β_1_ and α_5_β_1_) and non-RGD-recognizing (e.g., α_3_β_1_, α_2_β_1_ and α_1_β_1_) integrins (Ruoslahti, 1996; LaFoya et al., 2018; Ludwig et al., 2021). Moreover, several microorganisms, including GBS (Bolduc and Madoff, 2007), display adhesins with RGD motifs that can bind host integrins (Johnson et al., 2011; Bonsor et al., 2015; Deshayes de Cambronne et al., 2021). For these reasons, we examined the possibility that RGD-binding integrins function as receptors for GBS colonization and invasion of enterocytes. First, we evaluated the number of cell-adhering bacteria in the presence of an RGD peptide and its cognate non-functional RGE control (Viela et al., 2019; Mathelié-Guinlet et al., 2020). Under these conditions, neither peptide had inhibitory effects on bacterial adhesion to Caco-2 cells over a wide dose range. In contrast, in positive control experiments, the RGD, but not the RGE, peptide inhibited adherence of *S. aureus* (Supplementary Figure 1). Inhibition of *S. aureus* adherence by the RGD peptide suggests that the low levels of α_5_ and α_V_ integrins that are detectable on Caco-2 cells might still be relevant for *S. aureus* binding. Alternatively, *S. aureus* might interact with RGD-binding α-integrins not investigated in the present study. Collectively, these data suggested that RGD-recognizing integrins are not involved in GBS-enterocyte interactions (Figure 1A).

Next, we analyzed the expression of a panel of integrin subunits using immunofluorescence microscopy (Beaulieu, 1992; Perreault et al., 1995; Lussier et al., 2000). By this technique, we readily detected the presence of α_3_ and α_2_ subunits (Figure 1B). Moreover, higher expression of α_3_ and α_2_ subunits in these cells, in comparison with other alpha subunits, was apparent after quantification of fluorescence intensity (Supplementary Figure 2). The β_1_ integrin subunit, which is usually associated with the α_3_ and α_2_ subunits, was also found to be expressed at high levels in Caco-2 cells (Supplementary Figure 3). Notably, β_1_ displayed a uniform distribution on the cell surface, whereas α_3_ and α_2_ were selectively expressed on areas of cell contact (Supplementary Figure 2). The different cell surface distribution of these subunits is not surprising, and might be explained by the ability of β_1_ to associate with a large number of α subunits, some of which might be present on Caco-2 cells, in addition to α_2_ and α_3_ (van der Flier and Sonnenberg, 2001; Hynes, 2002; Frantz et al., 2010). Collectively, these data indicate that enterocyte-like cells predominantly express α_3_β_1_ and α_2_β_1_ integrins, while RGD-recognizing integrins containing α_V_ and α_5_ subunits, as well as the collagen-binding α_1_ integrin, are present at considerably lower levels. These findings are in general agreement with previous studies conducted on human intestinal tissues (Beaulieu, 1992; Perreault et al., 1995; Lussier et al., 2000).

### Subcellular location of α_2_ and α_3_ integrin subunits

**Figure 2:**
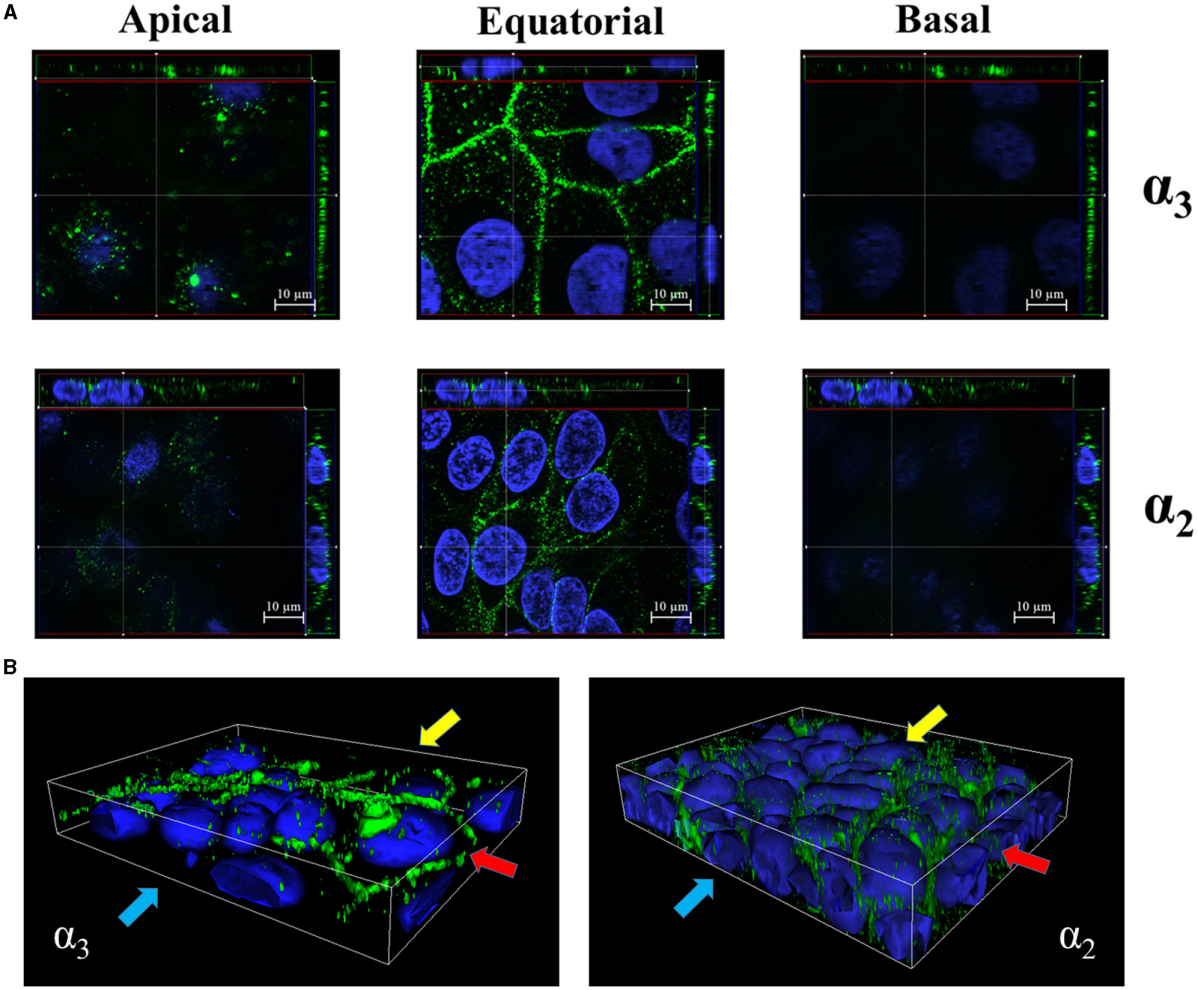
Equatorial localization of α_3_- and α_2_- integrin subunits in enterocyte-like cells. **(A)** *Z*-stack sections of 5-day-old Caco-2 cells with orthogonal views from *x*/*z* and *y*/*z* planes showing the distribution of α_3_- and α_2_- integrin subunits at the apical, equatorial and basal levels along the vertical axis of polarized enterocytes. Caco-2 cells were probed with antibodies specific to α_3_- and α_2_- integrin subunits followed by AlexaFluor 488- or FITC-labeled secondary antibodies (green), respectively, and analyzed by fluorescence microscopy. **(B)** 3D reconstruction of α_3_- and α_2_- integrin subunits distribution in 5-day-old Caco-2 cells. Eukaryotic nuclei and integrin subunits are visualized in blue (DAPI) and green, respectively, as described in **(A)**. Shown are images representative of three independent experiments. Yellow, red, and blue arrows indicate apical, equatorial, and basal planes, respectively, along the vertical cell axis.

Next, we studied the subcellular distribution of α_3_ and α_2_ integrins. Tri-dimensional structured illumination microscopy revealed that the α_3_ and α_2_ integrin subunits are preferentially expressed along the lateral surface of polarized Caco-2 cells and in the intercellular spaces, as indicated by orthogonal projections and 3D reconstructions (Figures 2A, B). Interestingly, both α_3_ and α_2_ integrins selectively clustered in the equatorial and parabasal segments of the lateral membranes in polarized cells, while low levels of these proteins were detectable in the apical and basal part of the lateral membranes (Figures 2A, B; Supplementary Figure 4). This pattern was not influenced by the degree of cell confluency in different observational fields (data not shown).

To determine whether α_3_β_1_ and α_2_β_1_ integrins are accessible to luminal agents in layers of polarized enterocytes, we performed ELISA assays on confluent Caco-2 monolayers obtained at 21 days post-seeding. Some monolayers were pretreated with EGTA, which is known to disrupt the integrity of intercellular junctions, before the addition of antibodies directed against α_3_, α_2_, and β_1_ integrin subunits. In the absence of EGTA, low-level binding of anti-integrin antibodies was detected, which increased considerably after cell exposure to the calcium-chelating agent (Supplementary Figure 5), suggesting that the accessibility of α_2_β_1_ and α_3_β_1_ integrins is increased after weakening intercellular junctions. Taken together, these results indicate that α_2_β_1_ and α_3_β_1_ are selectively located at the equatorial level of the lateral surfaces of polarized enterocytes in areas that are not readily accessible to luminal agents in the presence of intact intercellular junctions.

### Role of integrins in GBS adhesion to Caco-2 cells

**Figure 3:**
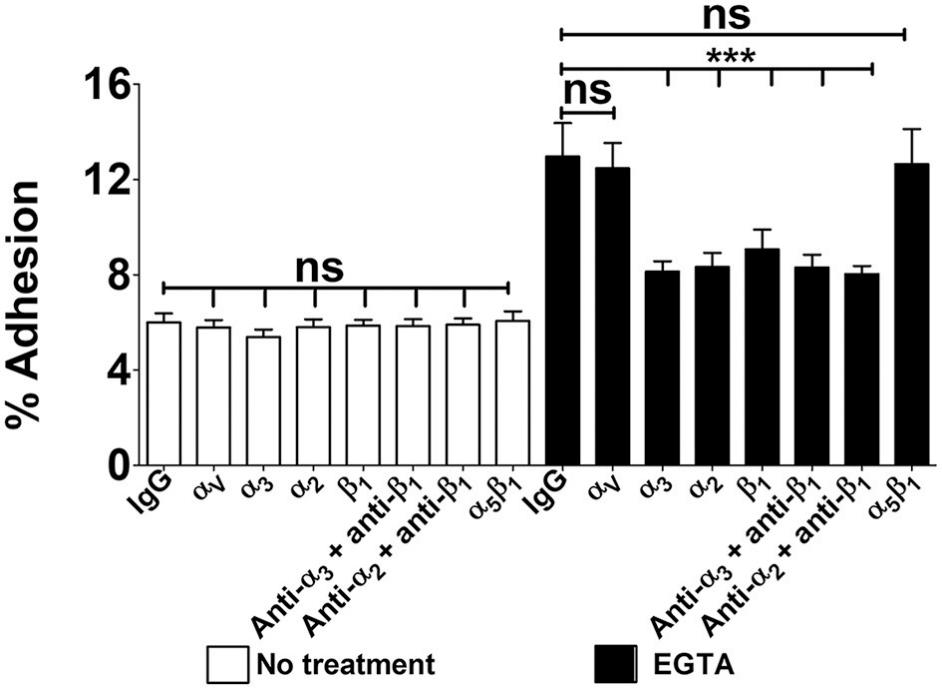
Integrin-dependent GBS adherence to confluent cell monolayers. Twenty-one-day-old Caco-2 cells were pre-treated with antibodies specific to α-, β-, or α/β integrin subunits (diluted 1:1,000) and infected with GBS. Streptococcal adherence is expressed as the percentage of input bacteria measured in cell lysates. IgG, normal IgG control. Shown are means ± SD of three independent experiments conducted in triplicate. Statistical analysis was performed by the ANOVA test followed by the Bonferroni correction, ns, non-significant; ***p < 0.001 compared to the normal IgG control.

**Figure 4:**
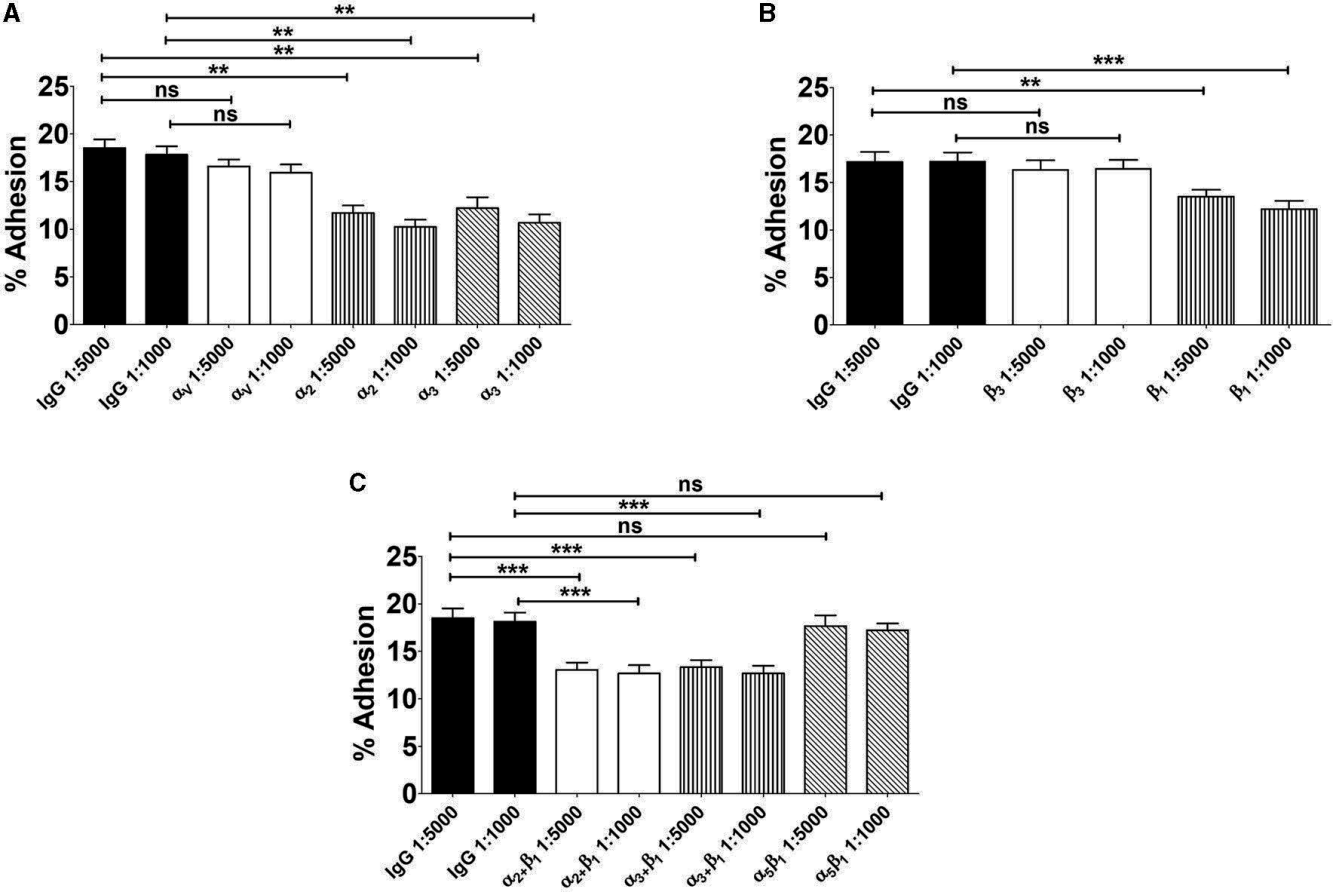
Involvement of α_3_β_1_ and α_2_β_1_ integrins in GBS adherence to enterocytes. Five-day-old Caco-2 cells were pre-treated with antibodies specific to α- **(A)**, β- **(B)**, or α/β **(C)** integrin subunits or normal IgG used as a control (IgG) at the indicated dilutions and infected with GBS. Streptococcal adherence is expressed as the percentage of input bacteria measured in cell lysates. Shown are means ± SD of five independent experiments conducted in triplicate. Statistical analysis was performed by the ANOVA test followed by the Bonferroni correction, ns, non-significant; **p < 0.01; ***p < 0.001 compared to the normal IgG control.

Based on the above findings we investigated the role of α_3_β_1_ and α_2_β_1_ integrins in GBS adhesion to enterocytes. In these experiments we used 21-day-old confluent cell monolayers treated with calcium-free saline to weaken intercellular junctions (De Gaetano et al., 2021). It was observed that cell pretreatment with antibodies directed against α_3_, α_2_, or β_1_ integrin subunits or mixtures thereof decreased GBS adherence, whereas anti-α_V_ or anti-α_5_β_1_ antibodies were ineffective (Figure 3). We further examined the effects of blocking antibodies using 5-day-old islands of polarized Caco-2 cells in which α_3_ and α_2_ integrins are freely accessible even in the absence of intercellular-junction-disrupting agents. Under these conditions, the numbers of adhering streptococci were significantly reduced after enterocyte pretreatment with anti-α_3_, anti-α_2_ or anti-β_1_ antibodies (Figures 4A, B), while mixtures of α_3_, α_2_ and β_1_ antibodies did not result in further reductions in GBS adherence compared to the values observed with single antibody types (Figure 4C). Collectively, these data indicate that α_3_β_1_ and α_2_β_1_ integrins promote GBS adherence to Caco-2 cells. However, our data also suggest the involvement of additional, as yet unidentified, receptors since only partial inhibition in bacterial adherence was observed using anti-integrin antibodies. It should be pointed out that we cannot formally exclude an involvement of the α_5_β_1_ integrin in GBS interactions with Caco-2 cells, based on our experiments, since we used only an anti-α_5_β_1_ monoclonal antibody in adherence inhibition experiments. Although effective at inhibiting bacterial adherence in a previous study using a different pathogen (Lentini et al., 2022), this monoclonal might recognize a region of the integrin that is not involved in interactions with GBS.

### Integrin α_3_β_1_ and α_2_β_1_ subunits mediate GBS invasion of Caco-2 cells

**Figure 5:**
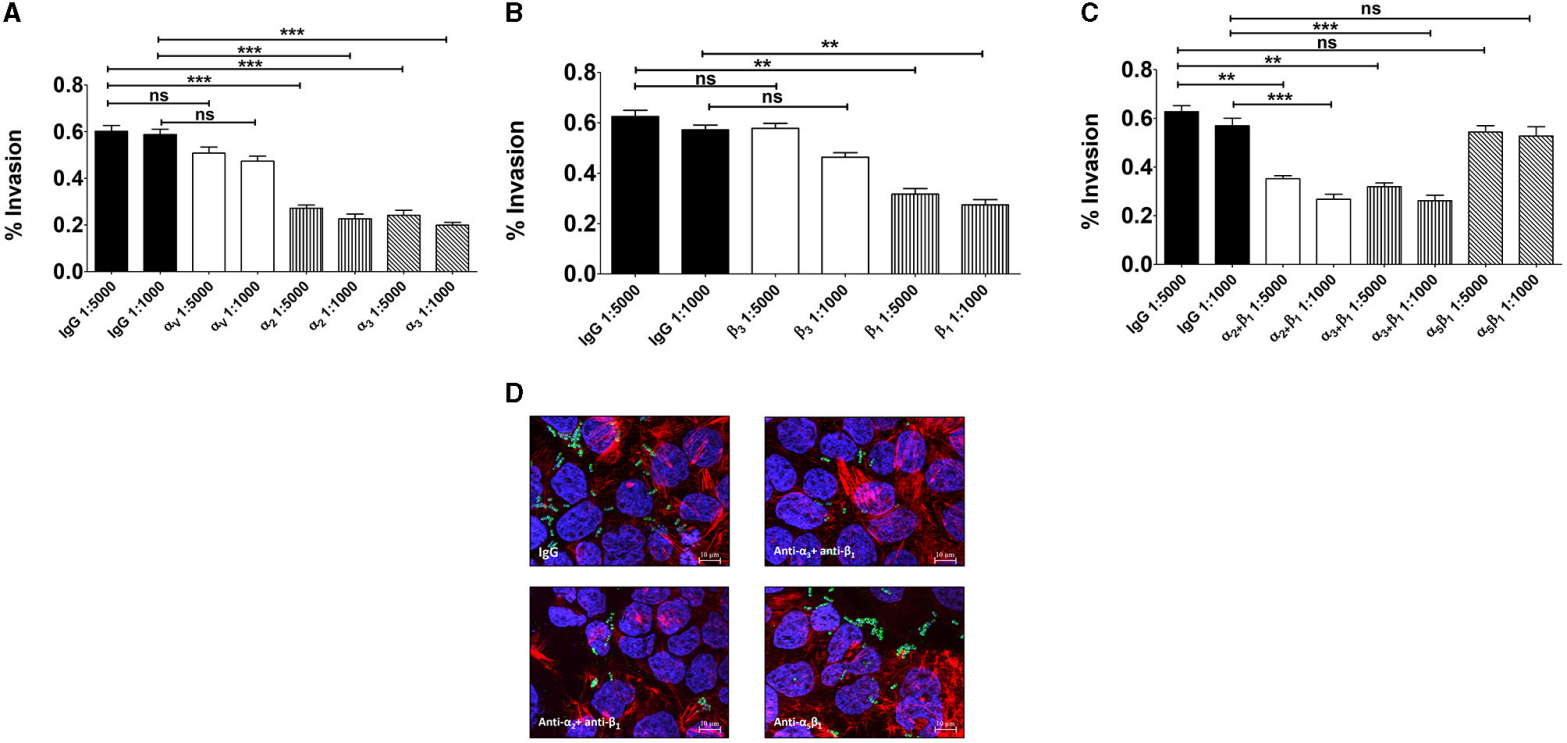
Integrins α_3_β_1_ and α_2_β_1_ are involved in GBS invasion of enterocyte-like cells. Five-day-old Caco-2 cells were pre-treated with antibodies to α- **(A)**, β- **(B)**, or α/β **(C)** integrin subunits or normal IgG used as a control (IgG) at the indicated dilutions and infected with GBS. Streptococcal invasion was measured after killing extracellular bacteria with antibiotics. Invasion is expressed as the percentage of input bacteria measured in cell lysates. Shown are means ± SD of five independent experiments conducted in triplicate. Statistical analysis was performed by the ANOVA test followed by the Bonferroni correction, ns, non-significant; **p < 0.01; ***p < 0.001 compared to the normal IgG control. **(D)** Effects of anti-α/β integrin subunits antibodies on GBS invasion as detected by immunofluorescence analysis of permeabilized cells. Nuclei and actin filaments were labeled with DAPI (Blue) and Phalloidin-iFluor 555 (Red), respectively. Bacteria (Green) were labeled using a rabbit anti-GBS serum followed by Alexa Fluor 488-conjugated anti-rabbit IgG. Images were obtained with the extended focus function. Scale bar = 10 μm.

**Figure 6:**
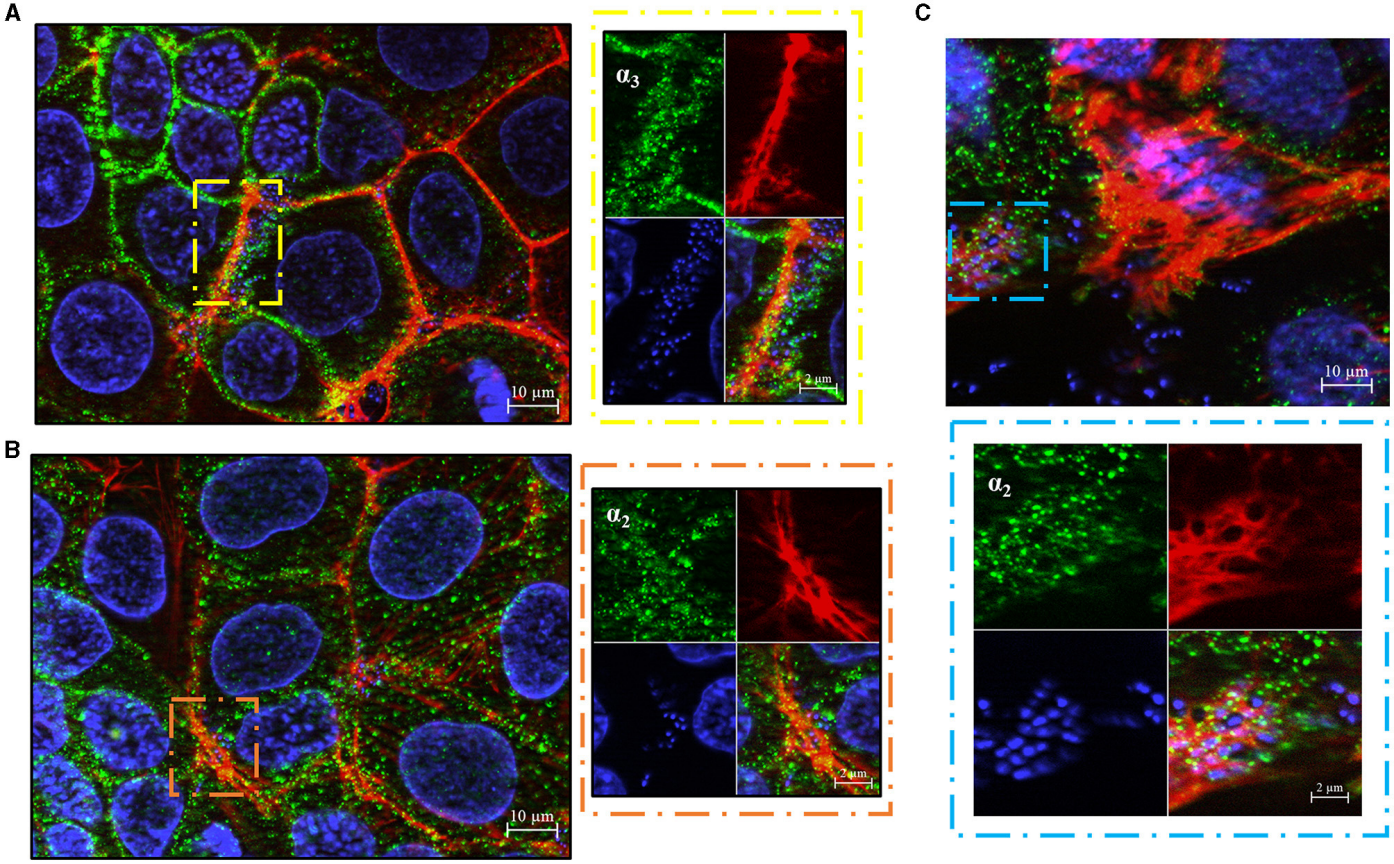
GBS trafficking in human enterocyte-cells. Immunofluorescence analysis of permeabilized 5-day-old Caco-2 cells showing spatial relationships between bacteria and α_3_- **(A)** or α_2_- **(B)** integrin subunits after bacterial internalization. Eukaryotic nuclei and bacterial nucleoids are labeled with DAPI (blue), while α_3_ and α_2_ integrin subunits are visualized in green using specific antibodies, by AlexaFluor 488- or FITC-labeled IgG secondary antibodies, respectively, and analyzed by fluorescence microscopy. The right panels are magnifications of the areas indicated by the dashed rectangles (yellow and brown). Scale bar = 2 μm. **(C)** Representative image showing streptococci inside actin-embedded vacuoles with α integrin subunits. Eukaryotic nuclei and bacterial nucleoids were labeled with DAPI (blue), actin filaments with Phalloidin-iFluor 555 (Red), α integrin subunit (green) with specific antibodies, followed by an AlexaFluor 488-labeled IgG secondary antibody. The lower panel is a magnification of the area indicated by the sky blue dashed rectangle. Scale bar = 2 μm.

We subsequently investigated whether the α_3_ and α_2_ integrin subunits could play a role not only in bacterial adherence, but also in GBS invasion of Caco-2 cells. To measure cell invasion in 5-day-old Caco-2 islands, we used an antibiotic protection assay in which extracellular bacteria are killed by antibiotics unable to penetrate the cell membrane. Under these conditions, pretreatment with anti-α_3_, anti-α_2_ and anti-β_1_, but not anti-α_5_β_1_, antibodies resulted in marked decreases in streptococcal internalization (Figures 5A–C). Notably, the extent of the antibody effects on internalization was higher than that previously observed on adherence. The involvement of α_3_β_1_ and α_2_β_1_ integrins in internalization was also confirmed by observing reduced numbers of intracellular bacteria after antibody treatment (Figure 5D). In these experiments we also observed a marked rearrangement of host cytoskeleton in areas of bacterial internalization. As shown in Figures 6A, B, the actin fibers clustered in areas in which α_3_ and α_2_ integrins, as well as streptococci, were present and bacteria were often seen inside intracellular spaces covered by actin and α_3_ and α_2_ integrin subunits (Figure 6C).

**Figure 7:**
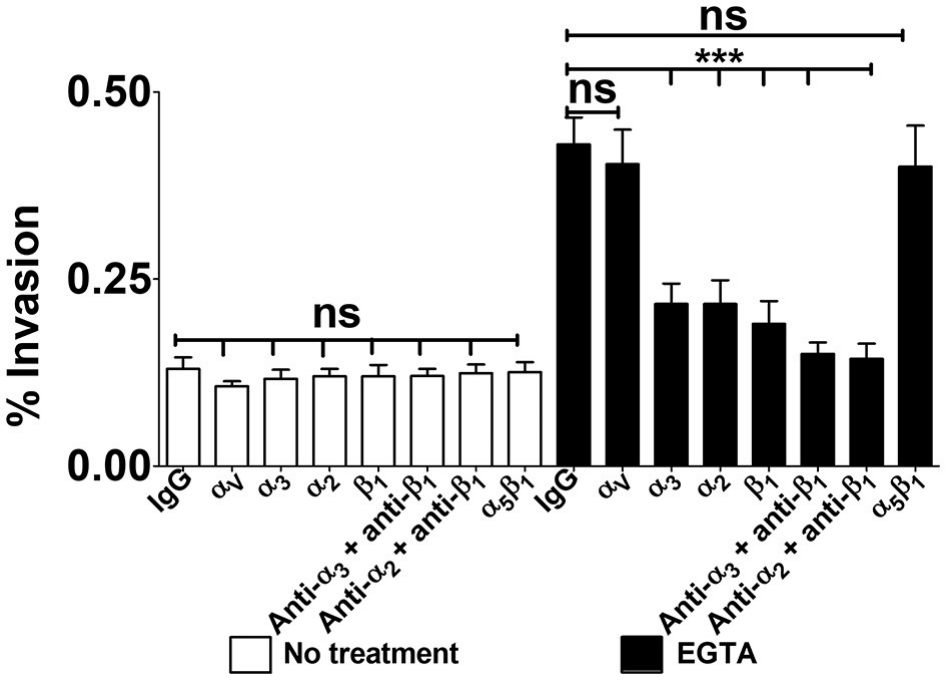
Integrin-dependent GBS invasion of confluent cell monolayers. Twenty-one-day-old Caco-2 cells were pre-treated with antibodies specific to α-, β-, or α/β integrin subunits or normal IgG control (IgG), all diluted 1:1,000, and infected with GBS. Antibiotics were then used to kill extracellular adherent bacteria. Invasion is expressed as the percentage of input bacteria measured in cell lysates. Shown are means ± SD of three independent experiments conducted in triplicate. Statistical analysis was performed by the ANOVA test followed by the Bonferroni correction, ns, non-significant; ***p < 0.001 compared to the normal IgG control.

To ascertain the involvement of α_3_β_1_ and α_2_β_1_ integrins in GBS invasion of confluent monolayers, Caco-2 cells were cultured for 2–3 weeks to obtain full cell polarization and differentiation. Next, monolayers were treated with EGTA in calcium free buffered saline, washed and incubated with anti-integrin antibodies before the addition of bacteria. As expected, the absence of Ca^++^ greatly promoted streptococcal uptake, and such an enhancement was completely abrogated in the presence of antibodies targeting α_3_, α_2_ or β_1_ integrin subunits (Figure 7). Moreover, treatment with EGTA was associated with increased accessibility of antibodies to α_3_β_1_ and α_2_β_1_ integrins (Supplementary Figure 5). All together, these data indicate that α_3_β_1_ and α_2_β_1_ integrins can function as receptors for GBS entry into polarized enterocytes through the equatorial and parabasal portions of their lateral surfaces.

## Discussion

A growing body of evidence suggests that the ability of CC17 GBS to colonize and invade the gut of newborns is relevant to pathogenesis of a large proportion of LODs (Tazi et al., 2010, 2019; Morinis et al., 2011; Hays et al., 2019; De Gaetano et al., 2021). However, little is known of the ability of GBS to colonize and invade the intestinal epithelium that represents an exceptionally robust physical barrier against any pathogen, due to the presence of specialized cell-to-cell junctions and the maintenance of apical-basal polarity (Snoeck et al., 2005; de Pereda et al., 2009; Franke, 2009). To study the interactions between hypervirulent GBS and enterocytes, we choose as a model the Caco-2 cell line which differentiates into monolayers that have many features of the intestinal epithelium (De Gaetano et al., 2021). We have previously shown that hypervirulent GBS adheres to the lateral surfaces of Caco-2 cells and then invades them by a caveolae-dependent internalization mechanism involving bacterial trafficking in acidified vacuoles (De Gaetano et al., 2021). However, the nature of the cell receptors mediating GBS invasion could not be identified. In the present study, we hypothesized that integrins may function as GBS receptors in enterocytes and examined their expression in Caco-2 cells.

We first observed that the degree of expression and subcellular location of the various integrin subunits is similar in human enterocytes and Caco-2 cells. For example, the higher expression in Caco-2 cells of α_3_ and α_2_, compared to α_1_, α_5_, or α_ν_, subunits is reminiscent of similar observations in intestinal tissue enterocytes (Beaulieu, 1992). In the latter, α_3_β_1_ and α_2_β_1_ integrins display a distinctive crypt-villus gradient by which α_3_ and α_2_ are almost exclusively expressed in villus and crypt enterocytes, respectively (Beaulieu, 1992; Perreault et al., 1995; Lussier et al., 2000). We found that both integrins are expressed on Caco-2 cells, suggesting that these cells have features of both immature and mature enterocytes. Notably, both the α_3_ and α_2_ integrins are selectively localized on the lateral surface of Caco-2 cells, frequently clustering at equatorial and parabasal levels along the vertical cell axis. Lack of integrin expression on the apical surface is also a feature of human enterocytes, in which α_3_β_1_ and α_2_β_1_ integrins are found exclusively on baso-lateral surfaces (Beaulieu, 1992). Although their functional role is unclear, these integrins may be expressed on lateral enterocyte surfaces to establish robust intercellular connections, since the α_2_β_1_ integrin was recently found to be required for stabilizing adherence junctions and barrier function in keratinocyte layers (Howden et al., 2021). Of note, the α_6_β_1_ integrin is selectively expressed on the basal surface of all tissue enterocytes (Perreault et al., 1995), but was not investigated in the present study. Future studies will examine the expression of α_6_β_1_ integrin on the basal surfaces of Caco-2 cells grown on plastic surfaces coated with different extracellular matrix components, including basal membrane components.

Because the distribution of α_3_ and α_2_ subunits corresponded to the area preferentially used by GBS to enter enterocyte-like cells, as determined in a previous study, we investigated here the role of these integrins. We first found that anti-α_3_ or -α_2_ antibodies induced significant inhibition of bacterial adherence, while antibodies directed against other α subunits were ineffective. Antibodies directed against the β_1_ chain, which is shared by the α_3_ and α_2_ subunits in the formation of heterodimeric complexes, were also inhibitory, suggesting the β_1_ chain contributes to bacterial adherence alone or when associated with its cognate α subunits. Notably, our data also suggest the involvement of additional, as yet unidentified, adhesion receptors since only partial inhibition of adherence to Caco-2 cells was observed using anti-α_3_, anti-β_1_, or anti-α_2_ antibodies. Future studies will be aimed at identifying these additional receptors as well as their cognate GBS adhesins. Interestingly, however, the anti-α_2_/α_3_/β_1_ antibodies used here were more effective at inducing inhibition of cell invasion than adherence suggesting a predominant role of the corresponding integrins in GBS internalization. For instance, bacterial invasion was considerably increased after opening cell-to-cell junctions in fully differentiated and polarized monolayers and such an increase was completely prevented using either anti-α_2_β_1_ or anti-α_3_β_1_ antibodies. These data are in agreement with our previous findings on the inhibitory effects of the tyrosine kinase inhibitor genistein on GBS invasion of enterocytes (De Gaetano et al., 2021), since the β_1_ subunit is known to activate a series of protein-tyrosine kinases that recruit actin and remodel the cell cytoskeleton (Lafrenie and Yamada, 1996; Shin et al., 2006; Ulanova et al., 2009; Cho et al., 2010). Moreover, our present data are in agreement with our previous ones on the importance of lipid rafts in GBS internalization, in view of the well-established role of these membrane domains in integrin signaling (Del Pozo, 2004). Accordingly, we observed here that integrins and actin filaments cluster in the same area of the cell around bacteria-containing vacuoles, which were previously identified as early and late endosomes (De Gaetano et al., 2021). Collectively, our studies indicate that GBS predominantly invade polarized enterocytes from their lateral surfaces using α_3_β_1_ and α_2_β_1_ integrins, which trigger a cellular response involving tyrosine-kinase dependent endocytosis and extensive actin remodeling. Interestingly, CC1 and CC17 GBS were previously shown to interact, respectively, with α_2_β_1_ and α_5_β_1/_α_v_β_3_ integrins in brain endothelial cells thereby promoting invasion of the blood-brain barrier (Banerjee et al., 2011). Of note, similar to what we found in enterocytes, integrins are normally expressed the basolateral and not apical surfaces of polarized endothelial cells (Bosman, 1993). Collectively these studies support a model whereby GBS must preliminarily induce alterations of some kind in continuous epithelial or endothelial layers in order to access integrins hidden in basolateral surfaces. GBS were previously shown to target intercellular junctions and to access paracellular spaces in epithelial cell lines monolayers (Soriani et al., 2006). Studies are underway to ascertain whether GBS-induced weakening of cell junction is mediated by cytolysins such as the granadaene pigment or the CAMP factor. It should be pointed out, however, that the role of α_3_β_1_ and α_2_β_1_ integrins in invasion of enterocytes by the CC17 BM110 strain, as shown here, cannot be generalized to GBS isolates belonging to other clonal complexes. Since CC17 GBS are known to express a unique set of adhesins and virulence factors (Lentini et al., 2018), future studies will be required to assess the role of various integrins using multiple clinically relevant GBS clone types.

Invasion of epithelial or endothelial cells from their basolateral surfaces, as shown here for GBS, is a common theme in the pathogenesis of bacterial and viral diseases (Seo et al., 2008; Bouwman et al., 2013) and in many cases integrins have been shown to participate in this process. For example, *Y. pseudotuberculosis* was found to interact with the α_5_β_1_ integrin along the basolateral membrane of enterocyte monolayers when epithelial cell junctions are transiently opened (McCormick et al., 1997; Kaur and Mukhopadhaya, 2020). Moreover, rotavirus can directly bind to the α_2_ integrin subunit on basolateral surfaces of polarized Caco-2 monolayers and penetrate cells by this route (Seo et al., 2008).

GBS is frequently responsible for fetal infection because of its ability to ascend from the vagina into the uterus and invade the amniotic cavity (Brokaw et al., 2021) and, in this context, increased exfoliation of GBS-colonized vaginal cells may promote bacterial migration (Vornhagen et al., 2018). It has been proposed that GBS interaction with the α_1_β_1_ integrin results in the upregulation of a series genes driving epithelial-mesenchymal-transition and epithelial exfoliation in vaginal cell and proinflammatory gene expression in brain endothelial cells (Banerjee et al., 2011; Vornhagen et al., 2018). Whether upregulation of these genes occurs in enterocytes upon engagement of α_2_β_1_ or α_3_β_1_ integrins will be the object of future investigations.

The nature of the GBS adhesins that might interact with α_3_β_1_ and α_2_β_1_ integrins is still unclear. In a previous study, a domain of the GBS alpha C protein was found to directly interact with the α_1_β_1_ integrin on the surface of a cervical epithelial cell line and to promote bacterial internalization (Bolduc and Madoff, 2007). Moreover, at least in the case of a ST-26 clinical isolate, the pilus tip adhesin PilA of GBS was found to promote interactions with collagen, which then functioned as a bridge to engage the α_2_β_1_ integrin (Banerjee et al., 2011). However, whether GBS belonging to frequent sequence types use this mechanism to interact with host cells, particularly brain endothelial cells, has been questioned (Dramsi et al., 2012). Future studies should ascertain whether GBS pilus proteins or other adhesins bind directly or indirectly α_2_β_1_ and α_3_β_1_ integrins on enterocytes and whether collagen or other extracellular matrix proteins are involved in these interactions. Collectively, however, our studies and those of others suggest that targeting integrins may represent an appealing strategy to prevent GBS disease.

## Data availability statement

The original contributions presented in the study are included in the article/Supplementary material, further inquiries can be directed to the corresponding author.

## Ethics statement

Ethical approval was not required for the studies on humans in accordance with the local legislation and institutional requirements because only commercially available established cell lines were used.

## Author contributions

GD: Conceptualization, Data curation, Investigation, Methodology, Writing – original draft. GL: Methodology, Writing – review & editing. FC: Methodology, Writing – review & editing. AF: Methodology, Writing – review & editing. GP: Conceptualization, Writing – review & editing. CB: Conceptualization, Supervision, Writing – original draft.

## Supplementary material

Supplementary Figure 1 *S. aureus* adherence is inhibited by the RGD tri-peptide. Five-day-old Caco-2 cells were pre-treated with increasing concentrations of RGD or RGE tri-peptides (ranging from 0.6 to 15 μM) and infected with *Staphylococcus aureus*, Newman strain. Bacterial adherence is expressed as the percentage of input bacteria measured in cell lysates. Shown are means ± SD of five independent experiments conducted in triplicate. Statistical analysis was performed by the ANOVA test followed by the Bonferroni correction, ns, non-significant; ^*^*p* < 0.05.

Supplementary Figure 2 Quantitative analysis of fluorescence in enterocytes stained for integrin subunits. **(A)** Five-day-old, fixed, non-permeabilized Caco-2 cells were probed with antibodies specific for α_5_-, α_1_-, α_V_-, α_3_-, α_2_, and β_1_-integrin subunits, followed by AlexaFluor 488- or FITC-labeled secondary antibodies (green), and analyzed by fluorescence microscopy. Normal IgG controls are shown in the lower panels. Cell nuclei were stained in blue with DAPI. Scale bar = 10 μm. Shown are representative images from three independent experiments. **(B)** Analysis of Integrated Density (IntDen), as performed with the Image J software on pictures obtained during the experiments described in **(A)**. IntDen values for normal IgG controls were 0.38 or lower. ^*^Significantly different from normal IgG controls; v significantly different from α_V_, α_5_ and α_1_ integrin subunits by the Mann-Whitney test (*p* < 0.05).

Supplementary Figure 3 Beta 1 integrin expression on 5-day-old Caco-2 cells. Fixed, unpermeabilized Caco-2 cells were probed with antibodies specific to the β_1_ integrin subunit, followed by AlexaFluor 488-labeled secondary antibody, and analyzed by fluorescence microscopy. Cell nuclei were stained in blue (DAPI) and the β_1_ integrin subunit was stained in green. Scale bar = 10 μm.

Supplementary Figure 4 Quantitative analysis of fluorescence along the vertical axis of polarized enterocytes stained for integrin subunits. *Z*-stack sections of 5-day-old Caco-2 cells with orthogonal views from *x*/*z* and *y*/*z* planes showing the distribution of α_3_- **(A)** and α_2_- **(C)** integrin subunits at the apical, equatorial and basal levels along the vertical axis of polarized enterocytes. Caco-2 cells were probed with antibodies specific to α_3_- and α_2_- integrin subunits, followed by AlexaFluor 488- or FITC-labeled secondary antibodies (green) and analyzed by fluorescence microscopy. Shown are representative images from five independent experiments. **(B, D)** Analysis of Integrated Density (IntDen) as performed with the Image J software on images obtained during the experiments described in A and C. ^*^Significantly different by the Mann-Whitney test (*p* < 0.05).

Supplementary Figure 5 Detection of α_3_, α_2_ and β_1_ integrin subunits in confluent cell monolayers. Expression of α_3_, α_2_, and β_1_ integrin subunits, as measured by ELISA, in 21-day-old EGTA-treated enterocytes is shown as fold change relative to the expression levels observed in 21-day-old non-EGTA treated monolayers. Integrin subunit expression on non-EGTA treated monolayers was normalized to the value of 1. Shown are means ± SD of three independent experiments conducted in triplicate. ^*^*p* < 0.05 by Mann-Whitney test.
