# Supplementary figures and images for: Engagement of α3β1 and α2β1 integrins by hypervirulent Streptococcus agalactiae in invasion of polarized enterocytes
Source: Front Microbiol. 2024 Mar 6;15:1367898. doi: 10.3389/fmicb.2024.1367898 (PMC10951081; doi:10.3389/fmicb.2024.1367898)

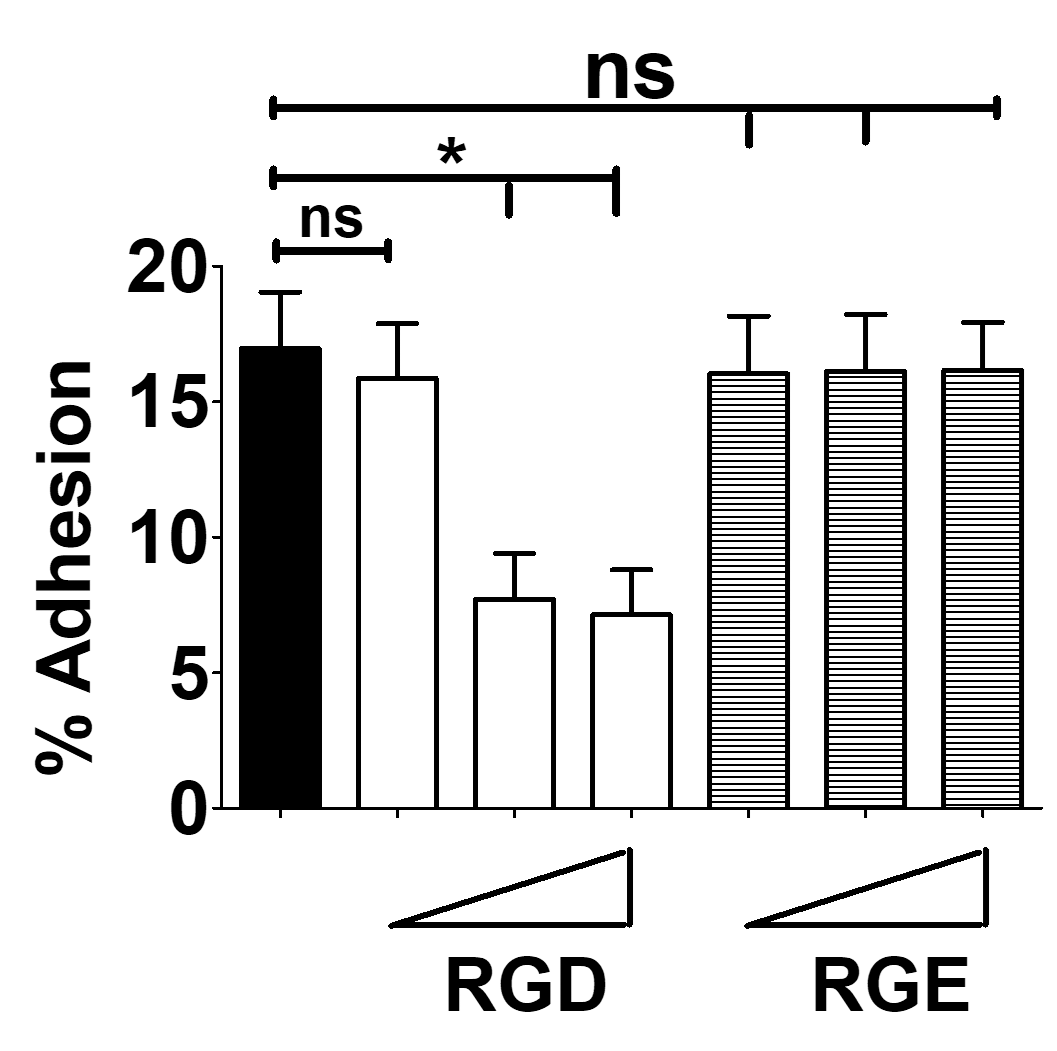

Supplement: Supplementary Figure 1 — S. aureus adherence is inhibited by the RGD tri-peptide. Five-day-old Caco-2 cells were pre-treated with increasing concentrations of RGD or RGE tri-peptides (ranging from 0.6 to 15 μM) and infected with Staphylococcus aureus, Newman strain. Bacterial adherence is expressed as the percentage of input bacteria measured in cell lysates. Shown are means ± SD of five independent experiments conducted in triplicate. Statistical analysis was performed by the ANOVA test followed by the Bonferroni correction, ns, non-significant; *p < 0.05. [file Image_1.TIF]

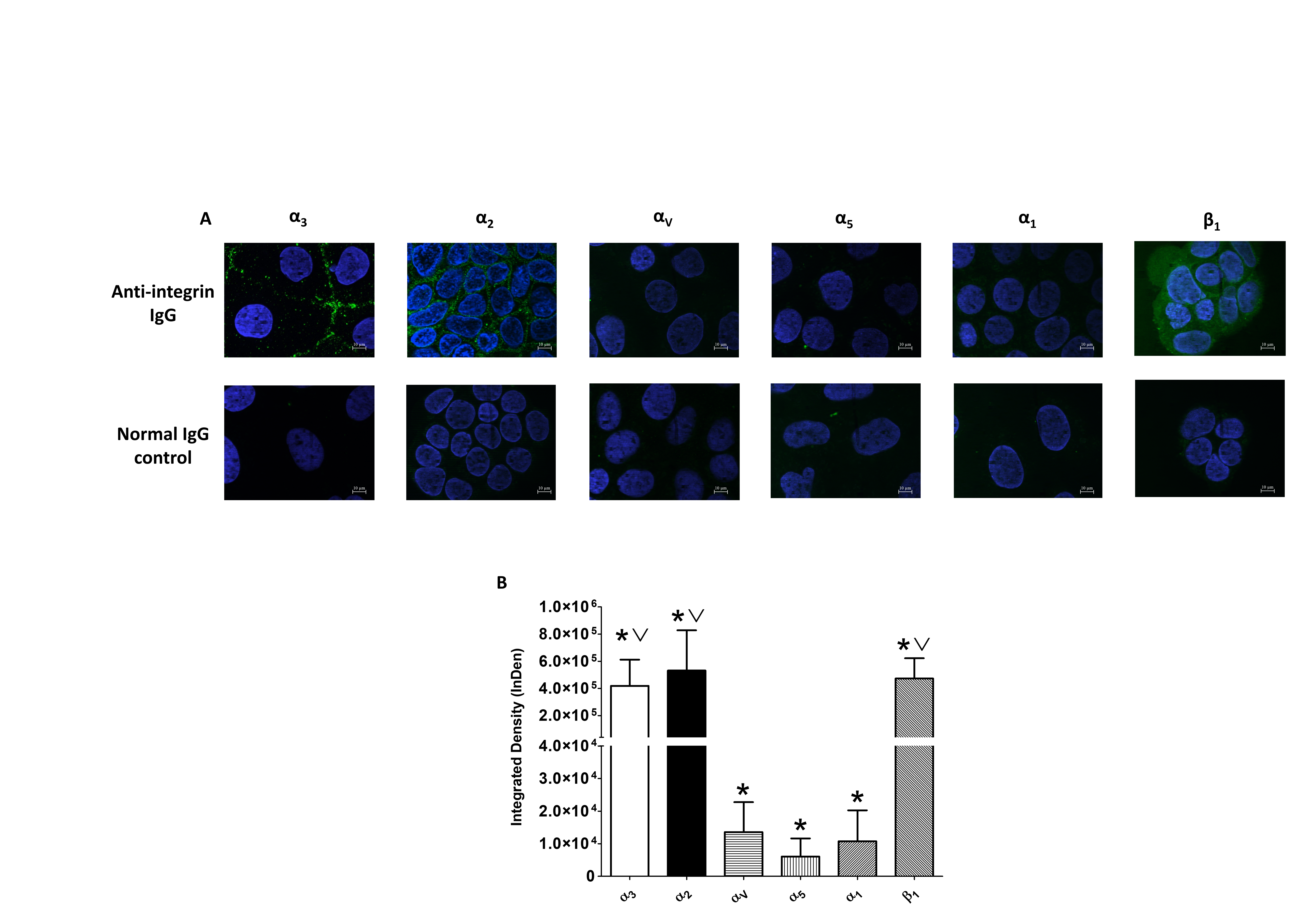

Supplement: Supplementary Figure 2 — Quantitative analysis of fluorescence in enterocytes stained for integrin subunits. (A) Five-day-old, fixed, non-permeabilized Caco-2 cells were probed with antibodies specific for α5-, α1-, αV-, α3-, α2, and β1-integrin subunits, followed by AlexaFluor 488- or FITC-labeled secondary antibodies (green), and analyzed by fluorescence microscopy. Normal IgG controls are shown in the lower panels. Cell nuclei were stained in blue with DAPI. Scale bar = 10 μm. Shown are representative images from three independent experiments. (B) Analysis of Integrated Density (IntDen), as performed with the Image J software on pictures obtained during the experiments described in (A). IntDen values for normal IgG controls were 0.38 or lower. *Significantly different from normal IgG controls; v significantly different from αV, α5 and α1 integrin subunits by the Mann-Whitney test (p < 0.05). [file Image_2.TIF]

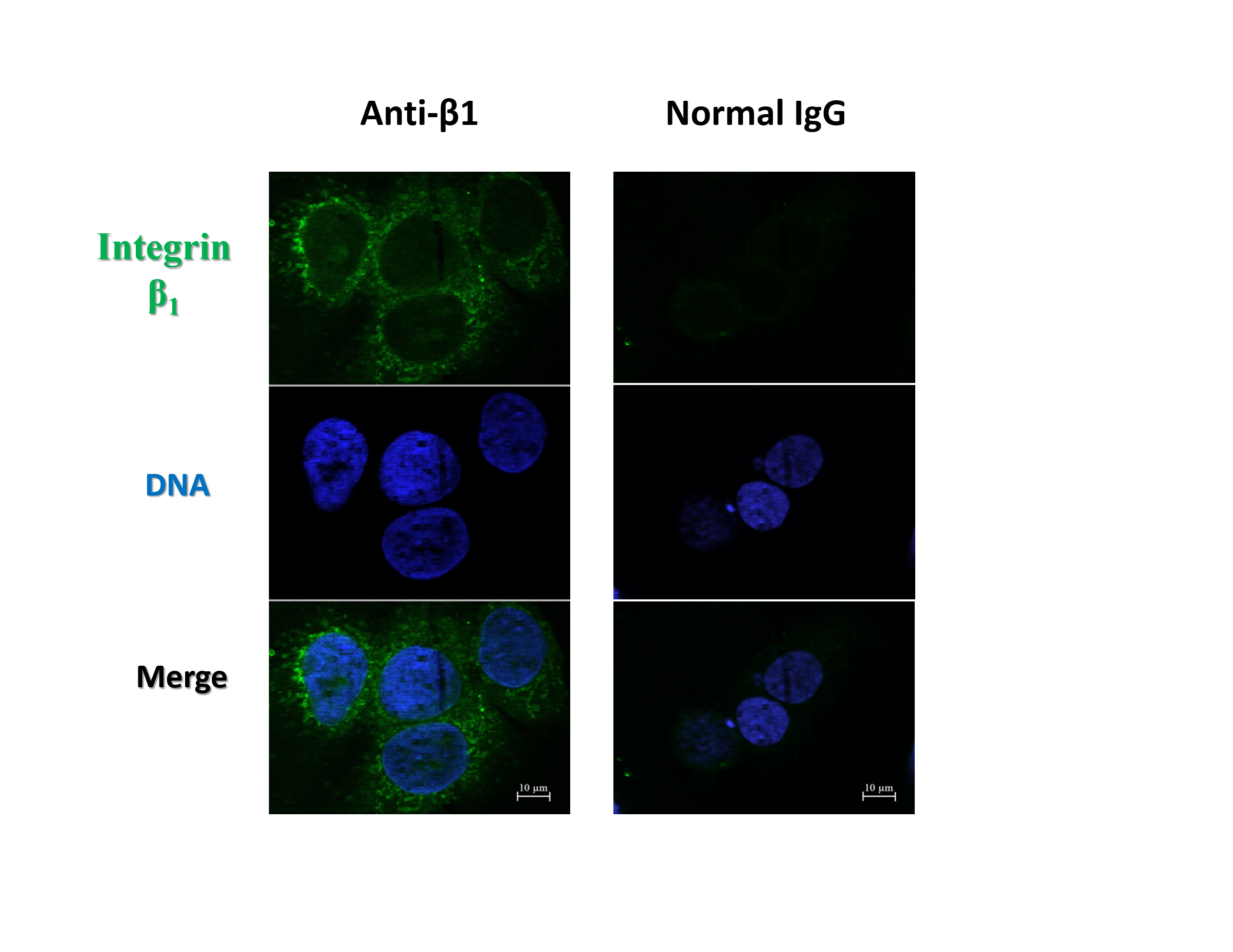

Supplement: Supplementary Figure 3 — Beta 1 integrin expression on 5-day-old Caco-2 cells. Fixed, unpermeabilized Caco-2 cells were probed with antibodies specific to the β1 integrin subunit, followed by AlexaFluor 488-labeled secondary antibody, and analyzed by fluorescence microscopy. Cell nuclei were stained in blue (DAPI) and the β1 integrin subunit was stained in green. Scale bar = 10 μm. [file Image_3.TIF]

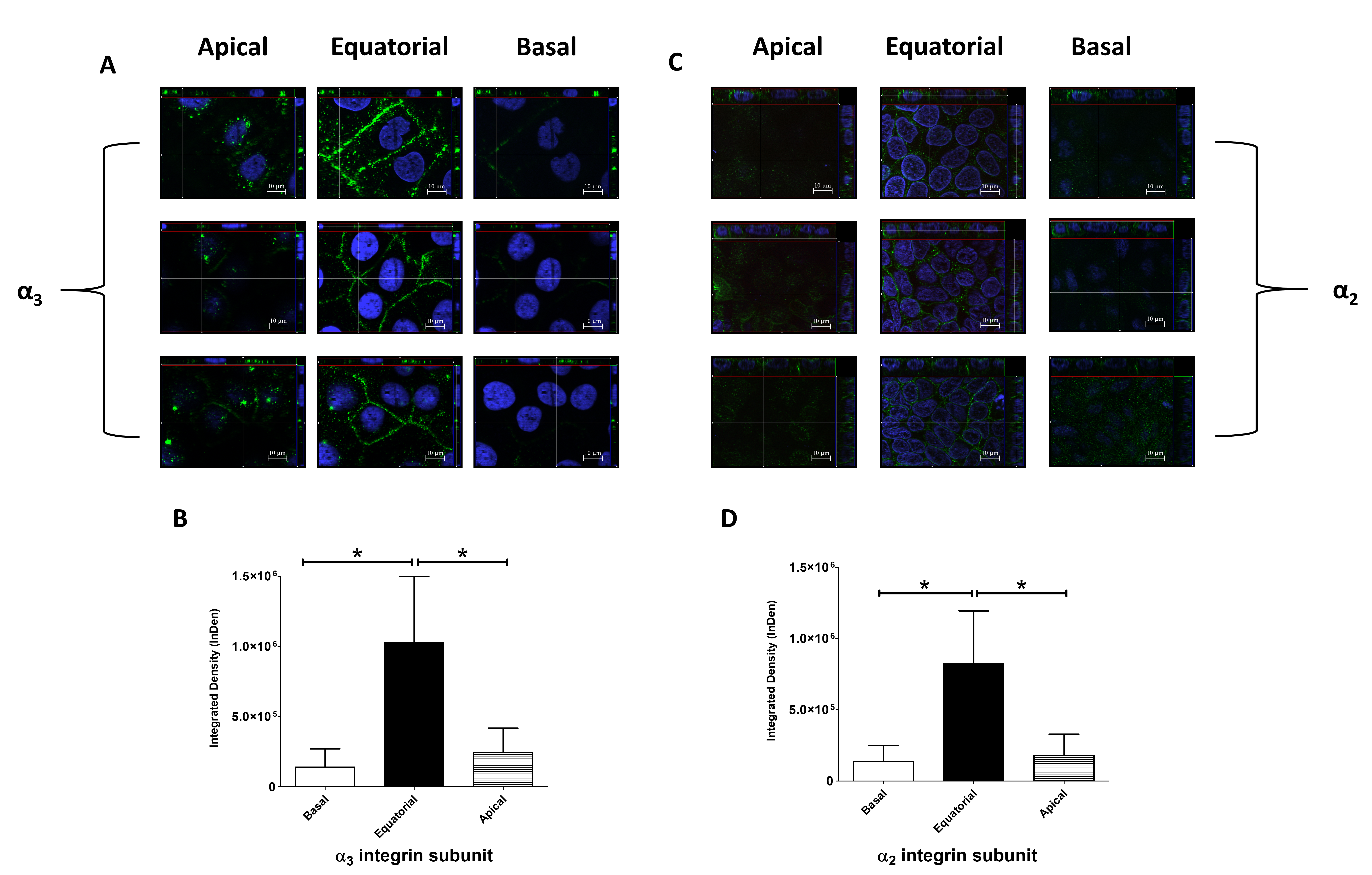

Supplement: Supplementary Figure 4 — Quantitative analysis of fluorescence along the vertical axis of polarized enterocytes stained for integrin subunits. Z-stack sections of 5-day-old Caco-2 cells with orthogonal views from x/z and y/z planes showing the distribution of α3- (A) and α2- (C) integrin subunits at the apical, equatorial and basal levels along the vertical axis of polarized enterocytes. Caco-2 cells were probed with antibodies specific to α3- and α2- integrin subunits, followed by AlexaFluor 488- or FITC-labeled secondary antibodies (green) and analyzed by fluorescence microscopy. Shown are representative images from five independent experiments. (B, D) Analysis of Integrated Density (IntDen) as performed with the Image J software on images obtained during the experiments described in A and C. *Significantly different by the Mann-Whitney test (p < 0.05). [file Image_4.TIF]

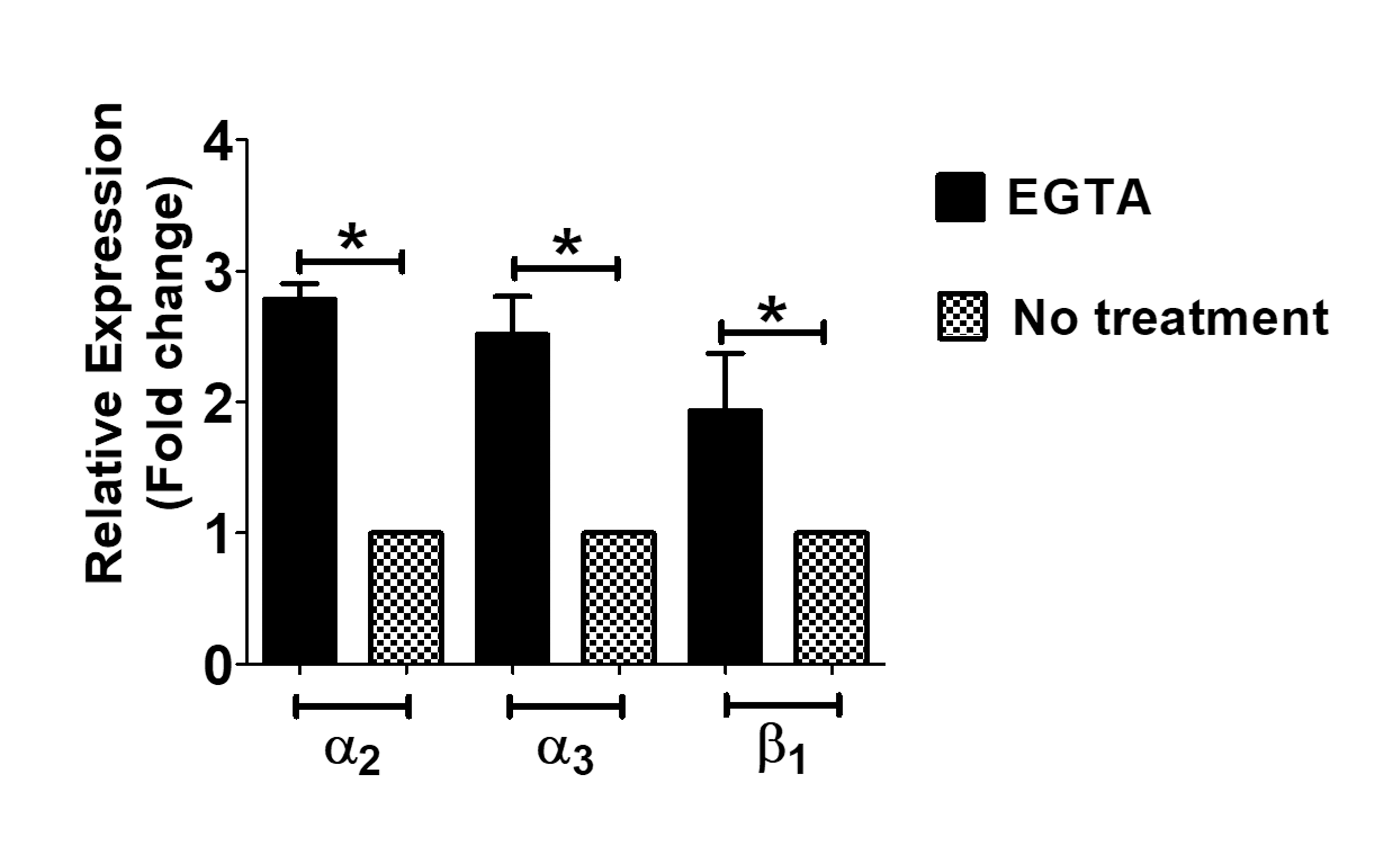

Supplement: Supplementary Figure 5 — Detection of α3, α2 and β1 integrin subunits in confluent cell monolayers. Expression of α3, α2, and β1 integrin subunits, as measured by ELISA, in 21-day-old EGTA-treated enterocytes is shown as fold change relative to the expression levels observed in 21-day-old non-EGTA treated monolayers. Integrin subunit expression on non-EGTA treated monolayers was normalized to the value of 1. Shown are means ± SD of three independent experiments conducted in triplicate. *p < 0.05 by Mann-Whitney test. [file Image_5.TIF]
